# Aortic valve calcification – the role of inflammation and fibrosis

**DOI:** 10.1186/1749-8090-8-S1-O32

**Published:** 2013-09-11

**Authors:** K Pissaridi, V Dritsa, J Anastassopoulou, E Koutoulakis, I Mamarelis, Chr Kotoulas

**Affiliations:** 1 Chemical Engineering Department, Radiation Chemistry & Biospectroscopy, National Technical University of Athens, Greece; 2 Department of Cardiology, NIMTS Veteran Hospital of Athens, Greece; 3 Department of Cardiology, 401 Army General Hospital of Athens, Greece; 4 Department of Cardiac Surgery, Iaso General Hospital of Athens, Greece

## Background

Pathogenesis of aortic valve calcificationis a multifunctional processassociated with various risk factors, while heart valve replacement is the only established treatment.Unfortunately, the real mechanism of the valve calcification remains still unknown. Herewith, we investigate the changes in molecular levelof aortic valve tissues in order to characterize the mineral deposits and understand the mechanism of aortic valve mineralization and stenosis.

## Methods

30 aortic valves samples of patients (65-80 years), who underwent surgical aortic valve replacement due to aortic valve stenosis. A Nicolet 6700 thermoscientific spectrometer was used to record the infrared (FT-IR) spectra. The aortic valve surfaces were studied with Scanning Electron Microscopy (SEM-EDX), without any coating of the samples.

## Results

The infrared spectra showed intensity changes and shifts of characteristic bands concerning the peroxidation of lipids suggesting inflammation character of stenosis. The proteins and collagen changed their α-helix to random one.The mineral deposits were consistent of calcium phosphates, CaHPO_4_ and amorphous hydroxyapatite, depending on the chemical factors of the cells’ microenvironment. In the case of SEM showed that the cross-link bonds of collagen are the targetingsites where the minerals start the deposition.

## Conclusions

The characteristic FT-IR absorption bands of calcified stenotic aortic valves showed hyperoxidation of membranes (a pro-inflammation stage), while the mineral deposits were consistent of low crystallinity biological HA (Ca_10_(PO_4_)_6_(OH)_2_), Ca_2_HPO_4_ and calcium phosphate of phospholipoprotein fragments. SEM-EDX data show substitution of calcium cations from magnesium cations leading to amorphous hydroxyapatites. This finding suggested that magnesium supplements could prevent the re-calcification of the implants.

